# Unveiling the Health Ramifications of Lead Poisoning: A Narrative Review

**DOI:** 10.7759/cureus.46727

**Published:** 2023-10-09

**Authors:** Taanvi Bhasin, Yashwant Lamture, Mayank Kumar, Rishi Dhamecha

**Affiliations:** 1 Surgery, Jawaharlal Nehru Medical College, Datta Meghe Institute of Higher Education and Research, Wardha, IND

**Keywords:** toys, child health, paint, lead, public health

## Abstract

The presence of lead (Pb) in children’s toys and paint is a significant global public health concern. Numerous studies conducted worldwide have measured lead concentrations in these products. This article aims to examine the research findings and shed light on the implications for human health, including legal consequences and public awareness. Despite regulations on lead levels in polyvinyl chloride (PVC) paints and children’s toys in many countries, several reviewed documents indicate that these products often contain substantial amounts of lead, frequently surpassing legal limits. Particularly high levels of lead in paints have been found in countries such as China, Thailand, and Brazil. It is crucial to raise awareness among parents by educating them about this issue and empowering them to take proactive measures to protect their children from lead poisoning associated with toys and colored paints. There is also global support for eliminating lead pigments and regulating the amount of lead in PVC toys.

## Introduction and background

Lead (Pb) is a naturally occurring toxic metal found on earth. Its extensive use has led to significant environmental pollution, human exposure, and serious public health concerns worldwide [1]. Activities like mining, manufacturing, and certain practices, including the continued use of leaded aviation fuel in select countries, contribute to air pollution with lead [2,3]. More than three-quarters of the global lead consumption is attributed to the production of lead-acid batteries for motor vehicles. However, lead has also been utilized in various other products, including pigments, paints, solders, colored glass, lead crystal glassware, ammunition, ceramic glazes, jewelry, toys, cosmetics, and even some traditional medicines, as outlined by legally binding measures on lead paint by the World Health Organization (WHO). Water transported through lead pipes can also carry lead contaminants [4,5].

Children are particularly vulnerable to the detrimental effects of lead exposure, which can have long-term health consequences, especially in terms of brain and central nervous system (CNS) development. Lead can cause severe harm in adults, including an increased risk of high blood pressure and kidney damage. Elevated levels of lead exposure in females can result in miscarriage, stillbirth, premature birth, and low birth weight infants, as highlighted in the press release regarding the “end of leaded gasoline.” The historical use of lead can be traced back to ancient times due to its essential physicochemical properties. While lead possesses valuable and indispensable characteristics such as softness, malleability, flexibility, low conductivity, and corrosion resistance, its persistent use poses increasing risks to the environment due to its adverse nature and its continued prevalence in various applications [6-8].

## Review

Lead (Pb) exposure in older homes primarily results from lead-based paints, which were a significant source of lead intoxication in children. In the late 1970s, the United States of America (USA) banned lead paint for residential use. Additionally, lead was once a component of gasoline and was released into the environment through vehicle emissions. Lead contamination in adolescents is often attributed to deteriorating paint, household dust, and contaminated soil in proximity to older residences, roads, airports, and industrial areas. Children are especially vulnerable to lead poisoning because they can absorb four to five times more lead from the gastrointestinal tract than adults, regardless of the source. Table 1 illustrates the two types of lead poisoning.

**Table 1:** Types of lead poisoning Created by the author

Type of poisoning	Exposure	Lead levels (μg/dl)	Clinical symptoms
Acute poisoning	Intense exposure of short duration	100-120	Muscle pain, fatigue, abdominal pain, headache, and vomiting
Chronic poisoning	Repeated low-level exposure over a prolonged period	40-60	Persistent vomiting, encephalopathy, lethargy, delirium, and coma

Methodology

In our quest to compile a comprehensive body of research, we meticulously accessed and mined various reputable databases renowned for their wealth of scientific literature. These databases, namely PubMed, Cochrane Library, and Google Scholar, served as the bedrock of our data acquisition. We searched specific keywords meticulously chosen to align with the focus and scope of our study. These keywords included “lead,” “poisoning,” “pathology,” “treatment,” “prevalence,” and “prevention.” 

Our search strategy was not confined to the mere identification of relevant articles through keyword queries; it extended further to encompass a meticulous manual examination of the reference lists appended to pertinent articles. This additional step was undertaken to unearth any potentially overlooked studies, ensuring a comprehensive and exhaustive survey of the available literature. 

By adopting this rigorous and multifaceted approach, we sought to assimilate a robust collection of research findings and insights into the multifarious facets of lead poisoning. The tool used for quality assessment was the Newcastle-Ottawa Scale. The total number of articles screened was 65, out of which 37 articles were used for this narrative review. This methodological rigor serves as the cornerstone of our study, underpinning the validity and comprehensiveness of our findings and contributing to the advancement of knowledge in the field of lead poisoning research.

Discussion

Lead exposure pathways in adolescents are diverse and encompass various sources. It can ensue from contact with objects such as vintage toys, jewelry, ceramics, and cosmetics, all of which may harbor lead constituents. Additionally, exposure can arise from the consumption of water that has traversed antiquated lead pipes or has been joined with lead-based materials. Imported confectionery and plant products have also been identified as potential sources of lead contamination [9]. Moreover, the innate curiosity of children, coupled with their proclivity to explore their surroundings orally, predisposes them to ingest items containing or coated with lead. This may include the inadvertent ingestion of soil contaminated with lead or the consumption of deteriorating paint chips containing lead, as highlighted in reports from the Institute for Health Metrics and Evaluation [10]. Over time, the body accrues lead in dental and skeletal tissues, with the subsequent release of lead from bones into the bloodstream during pregnancy, potentially exposing the developing fetus. Notably, malnourished children are particularly susceptible to lead toxicity, as their bodies exhibit an enhanced capacity to absorb lead in the absence of essential nutrients such as calcium (Ca) or iron (Fe) [8].

The demographic most vulnerable to the adverse effects of lead toxicity comprises young individuals, including developing fetuses, and those hailing from socioeconomically disadvantaged backgrounds. The ramifications of lead poisoning on children’s health are profound, manifesting as detrimental impacts on the CNS and the brain. These effects encompass symptoms ranging from fatigue and seizures to, in severe instances, mortality [11]. Even when exposure occurs at lower levels that do not yield immediate symptomatic presentations, lead’s pernicious influence extends across a spectrum of physiological systems. It can impede normative neurodevelopment in children, culminating in diminished intelligence quotient scores and behavioral alterations. These behavioral shifts may encompass reduced attention spans, tendencies toward antisocial behavior, and compromised academic performance, as outlined in assessments conducted under the Strategic Approach to International Chemicals Management (SAICM).

It is paramount to underscore that a substantial cohort of children afflicted by lead poisoning may remain asymptomatic, and even modest levels of lead exposure can precipitate cognitive and behavioral challenges, including deficits in attention regulation [12].

Lead Poisoning Symptoms Include

Table 2 highlights various symptoms associated with lead poisoning. In rare cases, extremely elevated lead levels can lead to disorientation, seizures, coma, and, exceptionally, even death. Babies who are exposed to lead while in the womb due to maternal exposure may also experience learning and developmental difficulties, as noted by the United Nations Environment Programme’s The Lead Campaign.

**Table 2:** List of symptoms seen in lead poisoning

Early symptoms	Late symptoms
Fatigue	Memory problems
Headache	Nausea
Irritability	Kidney problems
Metallic taste	Weight loss
Uneasy stomach	Constipation
Poor appetite	Weak wrists or ankles
Weight loss	
Reproductive problems	

Absorption of lead

Lead may enter the body through the lungs by inhalation, through the skin, or by direct swallowing and ingestion [9]. Inorganic lead absorption takes place throughout the respiratory and gastrointestinal tracts. For adults with occupational exposure, the most significant route for absorption is through the respiratory tract [2,9]. Respiratory lead absorption is primarily dependent on particle size. The percentage of inhaled lead reaching the bloodstream is estimated to be 30-40% [2]. Rates of absorption through the gastrointestinal tract depend on the nutritional status and the age of the individual exposed. Therefore, while adults absorb an average of 10-15% of the ingested quantity, this amount can increase to 50% in infants, young children, and pregnant women [2,9]. Absorption through the gut is the predominant route for children and increases when dietary intakes of iron, calcium, phosphorus, or zinc are low [2,9,10]. There is little transcutaneous absorption of lead when inorganic lead compounds, such as those found in paint, are applied to the skin. In contrast, organic (tetraethyl) lead, which is found in gasoline, can be absorbed via the skin. This route may have contributed to lead poisoning in chemical workers during the development of this gasoline additive in the 1920s [2,9,11].

Distribution of lead after absorption

Following exposure to lead, the element is absorbed into and transported by the bloodstream to other tissues. Once absorbed, lead accumulates in three compartments: blood, soft tissues, and bone. In blood, approximately 99% of the lead is found in the erythrocytes, leaving about 1% in the plasma and serum [12]. The concentration of lead in plasma is more significant than that in whole blood as the means of distribution to target organs, i.e., the brain, lungs, spleen, renal cortex, aorta, teeth, and bones [2,13]. The kinetics of lead transfer from blood to soft tissues are low and take approximately four to six weeks [2]. Lead in blood has an estimated half-life of 35 days [14]; in soft tissue, 40 days [15]; and in bones, 20-30 years [16]. The biological half-life of lead may be considerably longer in children than in adults [15]. Blood lead concentrations reflect the intake of only the previous three to five weeks and thus cannot be used as indices of chronic exposure [2,9,12]. The initial distribution of lead throughout the body is dependent on blood flow to the tissues. More than 95% of lead is deposited in skeletal bone as insoluble phosphate [2]. Autopsy studies have shown that 90-95% of the body’s burden is present in cortical bone and teeth. In adults, some 80-95% of the total body burden of lead is found in the skeleton, compared with about 73% in children. The residence period of lead in bone is up to 30 years, with lead concentrations in bone and teeth increasing as a function of age. Bone lead may be regarded as two physiologically distinct pools: an inert pool with a half-life of decades and a labile pool that readily exchanges with the lead present in blood or soft tissues [2,12]. It has been determined that lead crosses the placental barrier with fetal uptake, beginning at 12 weeks gestation and continuing throughout development up to birth. Concentrations of lead in umbilical cord blood were found to be 80-100% of the maternal blood lead level [2,17,18]. Several conditions known to increase bone turnover, such as pregnancy, lactation, chemotherapy, tumor infiltration of the bone, or postmenopausal osteoporosis, may be associated with the mobilization of lead in bone stores, leading to chronic lead toxicity [18-21]. Although hyperthyroidism also increases bone turnover, it has only rarely been implicated in the pathogenesis of lead poisoning, with only two reported cases [19].

Excretion of lead

Inorganic lead is not metabolized; however, alkyl lead compounds are oxidized by the hepatic P450 system. Generally, lead excretion is low, with the most significant route being via the urinary tract. The use of chelating agents can enhance lead excretion in urine, and this constitutes the basis of the therapeutic approach to lead poisoning. Lead may also be excreted with bile through the gastrointestinal tract. Although minute amounts of lead are excreted through the sweat and the nails, these routes do not have any practical significance. In general, lead is excreted extremely slowly from the body, with its biological half-life estimated at 10 years, thus facilitating accumulation in the body [2].

Effect of lead on different systems of the body

In this section, we will talk about how lead affects the body’s different parts. 

Effect on the Central Nervous System

Regarding the most susceptible target for lead (Pb)-induced intoxication, Pb’s primary impact is on the neurological system, with a pronounced tendency to result in cognitive impairment. In adults, peripheral nervous system issues are highly probable, while in children, CNS involvement is more common [4,16]. These effects can manifest in both morphological and pharmacological ways. Morphological effects, particularly during prenatal and neonatal periods, disrupt neural tissue development, impeding processes like neuronal migration and differentiation, synaptic function, sialic acid synthesis in neurons, and early glial cell differentiation. Pb interferes with calcium (Ca) and zinc (Zn) metabolism, activating a calmodulin-dependent pathway that disrupts cellular calcium regulation, leading to the production of reactive oxygen species and cellular damage [17].

The impact of Pb on other bodily systems that influence nervous system function can have unpredictable consequences for the CNS. Research indicates that exposure to high levels of Pb exacerbates conditions such as hypertension, kidney failure, thyroid dysfunction, vitamin B deficiency, and preterm delivery [5]. Prolonged exposure to Pb can result in cognitive decline and even mortality. Among individuals exposed to toxic substances at work, peripheral neuropathy is the most common condition [6,18].

*Effect on the Renal System* 

Elevated levels of lead exposure have been consistently associated with a range of kidney abnormalities, even when lead concentrations are relatively modest, such as around 10 µg/dl [7,19]. Of particular concern is the impact on children, where early-stage kidney disease is more frequently observed, and it tends to be potentially reversible in this age group [8]. The pathophysiology of lead-induced kidney disease involves complex mechanisms. Acute kidney injury due to lead exposure often involves regressive changes in the tubular epithelium, where lead accumulates in the form of nuclear envelopes containing lead-protein complexes. These alterations in the renal tubules can lead to functional impairments and represent key features of acute kidney disease [8,20].

In contrast, long-term lead-induced nephropathy is more prevalent among older individuals, and it typically progresses irreversibly [5,21]. This condition is characterized by extensive damage to glomerular and medullary tissues within the kidneys. As a consequence, individuals with lead-induced nephropathy often exhibit elevated uric acid levels, hypertension (high blood pressure), and, over time, a heightened risk of kidney failure [6]. These findings underscore the insidious and potentially lifelong consequences of lead exposure on renal health, with a particular vulnerability observed in children. It reinforces the importance of robust preventive measures to limit lead exposure, especially among the most vulnerable populations, to mitigate the risk of kidney-related health issues.

Effect on the Cardiovascular System

Lead’s impact on the cardiovascular system extends beyond merely causing hypertension. It encompasses a spectrum of adverse effects on various cardiovascular parameters, including chronic heart disease (CHD), stroke, peripheral artery disease, and an array of cardiovascular functional abnormalities. These encompass conditions like coronary heart disease, left ventricular hypertrophy, and alterations in heart rate [22]. To elucidate the mechanisms underlying lead’s cardiovascular effects, two primary modes of action come into focus. Firstly, lead exerts influence on the renin-angiotensin system, a critical regulator of vascular smooth muscle contraction. Secondly, lead’s interference with calcium utilization within the kidneys has repercussions on renal output regulation [3]. Lead exposure can induce nephropathy, a condition characterized by kidney damage that tends to persist over the long term, primarily observed in adults. Significantly, the effects of lead-induced nephropathy are generally irreversible [5].

These findings emphasize the intricate interplay between lead exposure and cardiovascular health, illustrating that lead’s impact extends to a spectrum of cardiovascular disorders and is rooted in its multifaceted influence on physiological systems, including the renin-angiotensin system and renal function. Recognizing these mechanisms is vital for developing strategies to mitigate lead’s detrimental effects on cardiovascular well-being.

Effect on the Reproductive System

The reproductive systems in males and females have been concerned with the aid of Pb [4]. Some studies show that decreased somatic development in kids is related to excessive blood Pb levels [5]. Reduced intercourse drive, sperm formation (decreased motor skills and numbers), chromosome destruction [23], infertility, abnormal prostate gland functioning, and changes in serum testosterone [24] are all negative consequences for male reproductive tissues. Lead’s effect on a woman’s reproductive tissues is more noticeable, such as premature birth, low birth weight, and advancing issues in childhood [25].

Effect on the Gastrointestinal System

In cases of prolonged lead (Pb) poisoning, individuals may experience severe abdominal pain characterized as dry colic, which occurs in conjunction with chronic constipation. This condition is often accompanied by a loss of appetite and an unpleasant metallic taste in the mouth. Notably, colon issues and constipation manifest as relatively late and sudden complications of chronic Pb poisoning. Prior to the onset of these gastrointestinal symptoms, individuals may experience sweating and vomiting as early warning signs [8,26].

Effect on the Respiratory System

While some studies have uncovered a connection between blood lead (Pb) levels and respiratory issues in children, it’s important to note that blood Pb levels are not strongly associated with asthma diagnoses [27]. However, elevated blood lead levels can exacerbate asthma symptoms in children, possibly due to increased levels of eosinophils and immunoglobulin E (IgE) [28]. This highlights the potential role of lead exposure in influencing the severity of asthma in pediatric populations.

Prevention and treatment

Preventing lead poisoning is of paramount importance due to its severe consequences, as once lead enters the body, complete removal or reversal of its detrimental effects is exceedingly challenging [29-32]. To address this public health concern comprehensively, a three-pronged preventive approach encompassing individual intervention, preventive medicine strategy, and public health strategy has been proposed [33].

Individual Intervention

This facet focuses on early identification of individuals, especially children, at high risk of lead exposure. Key components include regular screening of blood lead levels. If elevated lead levels are detected, prompt medical intervention is initiated with the primary goals of controlling adverse effects and preventing further lead accumulation.

Preventive Medicine Strategy

This complements individual-level efforts and operates on a broader scale. It entails public health initiatives aimed at reducing the risk of lead exposure within inhabited areas. Measures include stringent zoning regulations to prohibit the establishment of lead-related industries in close proximity to residential areas. Additionally, it emphasizes the complete substitution of lead with suitable alternatives where available.

Public Health Strategy

Operating at a population level, this strategy seeks to minimize the risk of lead exposure within habitable regions. Public health services advocate for a range of preventive measures, including stringent prohibitions against the setup of lead-related industries near residential zones and the complete elimination of lead when viable substitutes exist.

Furthermore, nutrition emerges as a crucial element in preventing lead-induced toxicity. Scientific studies have illuminated the protective role of specific nutrients, such as mineral elements, flavonoids, and vitamins, against environmental lead exposure and existing lead burden within the body. These nutrients play a pivotal role in restoring the delicate balance between prooxidants and antioxidants disrupted by oxidative stress. While the precise mechanisms behind this protective action are still under investigation, substantial data suggest that nutrients play a significant role in shielding against lead poisoning [32]. In conjunction with these multi-faceted preventive strategies, it is vital to underscore primary prevention through minimizing lead exposure. The gradual phasing out of leaded gasoline across numerous regions, alongside various lead reduction initiatives, has resulted in a significant reduction in population-level blood lead levels, exemplifying the positive impact of comprehensive prevention efforts. Nevertheless, continued dedication is essential, particularly in the eradication of lead-based paint, as only a minority of countries have legally mandated lead paint regulations [34].

The WHO recognizes lead as one of the top 10 elements of significant public health concern and underscores the responsibility of nations to safeguard the health of workers, children, and women of childbearing age. The WHO has made available a wealth of resources, including policy-relevant information, technical guidance, and advocacy materials, to support global efforts in addressing lead-related health issues [35].

*Treatment* 

The initial step is to do a confirmatory venous lead level examination. This should be done promptly if the screening result is >70 µg/dl, within 48 hours if the result is between 45 and 59 µg/dl, within one week if the result is 6-44 µg/dl, and within one month to three months if the result is <5 µg/dl [36]. A comprehensive environmental history should be taken by the doctor for children with lead levels of 15-19 µg/dl. Parents should be given advice on both preventive and good diet. Iron and calcium supplements, a low-fat diet, and frequent meals are all nutritional therapies that have been linked to lower gastrointestinal absorption of ingested lead [36]. If the confirmed blood lead levels remain between 15 and 19 µg/dl, blood lead levels should be tested again within two months. Individualized case treatment begins at blood lead levels of 20 µg/dl and comprises a complete medical history, nutritional evaluation, physical examination, environmental investigation, and hazard reduction. Chelation treatment may be advised; however, it is not generally advised at blood lead levels of 45 µg/dl [37].

## Conclusions

In conclusion, the WHO has undertaken a collaborative initiative with European business associations, policymakers, public health authorities, and healthcare professionals to establish precise specifications and comprehensive guidelines aimed at safeguarding both children and adults from the perils of lead poisoning. This strategic partnership recognizes that lead-based paint represents a substantial source of lead exposure in numerous countries. To combat this pervasive issue, a global alliance, jointly led by WHO and the United Nations Environment Program, has been established, bearing the collective mission of eradicating lead paint from our environments. The concerted efforts of this alliance are geared toward the formulation of clear objectives and the expeditious implementation of measures that align with our global aspirations: the prevention of childhood exposure to lead through paints and the overarching reduction of lead exposure originating from such sources. Lead poisoning, a multifaceted concern, exerts its detrimental effects across various physiological systems within the body, imposing adverse consequences on the gastrointestinal, neurological, and reproductive systems. In a significant disruption, lead impairs the customary deoxyribose nucleic acid (DNA) transcription pathway, ultimately manifesting as debilitating bone damage. It is imperative to underscore that lead serves no constructive structural role within the intricate framework of the human body. This review underscores the gravity of lead poisoning as a global public health challenge and emphasizes the pivotal role that international collaboration, as exemplified by WHO and its partners, plays in shaping policies and practices aimed at shielding individuals from the insidious consequences of lead exposure. As we navigate the path forward, it is our collective responsibility to remain committed to these objectives, safeguarding current and future generations from the enduring threat of lead poisoning.
